# Prevalence of Antiphospholipid Antibody Syndrome Among Patients with Recurrent Pregnancy Loss: Impact of the Revised 2023 ACR/EULAR Antiphospholipid Syndrome Criteria

**DOI:** 10.3390/jcm13247698

**Published:** 2024-12-17

**Authors:** Marion Mercier, Alain Lescoat, Morgane Pierre-Jean, Erwan Dumontet, Maela Le Lous, Nicolas Belhomme

**Affiliations:** 1 Department of Gynecology Obstetrics and Human Reproduction, University Hospital of Rennes, 35000 Rennes, France; marion.mercier@chu-rennes.fr (M.M.); maela.le.lous@chu-rennes.fr (M.L.L.); 2 Department of Internal Medicine and Clinical Immunology, University Hospital of Rennes, 35000 Rennes, France; alain.lescoat@chu-rennes.fr; 3 Inserm, EHESP, Irset (Institut de Recherche en Santé, Environnement et Travail)-UMR_S 1085, 35000 Rennes, France; 4 LTSI-Inserm UMR 1099, University of Rennes, 35000 Rennes, France; morgane.pierre.jean@chu-rennes.fr; 5 Laboratory of Immunology, University Hospital of Rennes, 35000 Rennes, France; erwan.dumontet@chu-rennes.fr; 6 Inserm-UMR U1236, 35043 Rennes, France

**Keywords:** antiphospholipid antibody syndrome, recurrent pregnancy loss, miscarriages, internal medicine, placental pathology

## Abstract

**Objectives:** Current guidelines recommend systematic screening for rheumatic diseases (RDs), including antiphospholipid syndrome (APS), in patients with recurrent pregnancy loss (RPL). However, these recommendations are based on limited evidence, as data on the prevalence of RD in this specific population remain scarce. In particular, the impact of the recent update to the ACR/EULAR classification criteria for APS on the prevalence of RD among RPL patients has yet to be clarified. To address these gaps, this study aims to (i) assess the impact of the 2023 ACR/EULAR APS classification criteria in patients with recurrent pregnancy loss (RPL); and (ii) analyze the prevalence of RD in these patients. **Methods:** We conducted a retrospective cohort study at Rennes University Hospital. From January 2010 to December 2021, all patients referred to the Clinical Immunology Department for RPL were included. Patients were eligible if they had undergone a full RPL evaluation, according to guidelines. **Results:** We included 165 women with RPL. APS according to the Sydney criteria was found in 24 (14.5%) patients. No significant differences in obstetric history or clinical signs were observed between APS-positive and APS-negative individuals. Only two patients fulfilled the updated 2023 APS criteria, resulting in 163 (98.8%) patients being classified as having unexplained recurrent pregnancy loss (uRPL). Among them, 108 had a new pregnancy following uRPL, resulting in 87 (81%) live births and 21 (19%) recurrent miscarriages. We did not identify any prognostic factor associated with subsequent pregnancy outcomes, including the patients’ antiphospholipid biological profile. We found a prevalence of non-APS RD of only 2.4% in the study population, including systemic lupus erythematosus, rheumatoid arthritis, and Behçet’s disease. **Conclusions:** APS was identified in 14.5% of the patients based on the former Sydney criteria and 1.2% according to the revised criteria. The lack of clinical differences between APS and non-APS patients aligns with previously reported limitations of the Sydney criteria in accurately identifying aPLA-related RPL. According to the rarity of APS as per the updated criteria, future large collaborative trials will be needed to further characterize APS-related RPL patients and to determine the best treatment strategy for future pregnancies.

## 1. Introduction

Recurrent pregnancy loss (RPL) is a major health issue, affecting two to five percent of couples, and is associated with maternal morbidity, psychological distress, and increased healthcare costs [1,2,3]. RPL is generally defined as three consecutive first-trimester pregnancy losses [4,5,6,7]. The recurrence risk of miscarriage in women with RPL ranges from 28.3% to 42.1% [8,9]. Identifying potential underlying causes of RPL is crucial to improving the prognosis of subsequent pregnancies.

A standard RPL work-up includes testing for antiphospholipid antibody syndrome [5]. In several countries, patients are routinely referred to rheumatologists or internal medicine specialists for clinical examinations to identify signs of rheumatic diseases (RD) and undergo antiphospholipid syndrome (APS) testing [10]. Antiphospholipid syndrome is a systemic autoimmune disease characterized by arterial and/or venous thrombosis and pregnancy morbidity associated with circulating antiphospholipid antibodies (aPLAs) [11].

According to the 2009 Sydney classification criteria, the clinical manifestations of obstetric APS (oAPS) include either three recurrent pregnancy losses (RPLs) before 10 weeks of gestation (WG), an unexplained intrauterine fetal death (IUFD) beyond 16 WG, or premature birth or early-onset pre-eclampsia before 34 WG in women with no history of thrombosis [11].

While the association between oAPS and placental-related fetal death [12,13] is well documented, the 2013 consensus report from the 14th International Congress on Antiphospholipid Antibodies underlined that only a few studies exploring the relation between APS and miscarriages actually met the classification criteria, making the association between RPL and aPLA questionable. Furthermore, while the combination of low-dose aspirin (LDA) and low-molecular-weight heparin (LMWH) increases the live birth rate from 10% to 80% in the subsequent pregnancies of women with oAPS with previous late fetal death [14,15], no therapeutic study has confirmed the efficacy of this treatment regimen in the specific subset of oAPS-RPL patients [10,16,17,18,19,20,21,22]. Thus, if the association between aPLA and fetal death or early onset pre-eclampsia is well documented [23,24], data supporting the role of aPLA in miscarriages are still controversial [25,26]. In 2023, new classification criteria were published by the ACR/EULAR [7]. Per these updated criteria, isolated RPL is no longer sufficient to meet the clinical criterion domain, and the new classification requires additional APS-related complications, including fetal death > 16 WG or pre-eclampsia, to be classified as APS [27].

The impact of the 2023 ACR/EULAR APS criteria on the management of patients with RPL, as encountered in routine clinical practice, is still to be determined. The aim of this study was to evaluate the results of an outpatient clinic specializing in rheumatology/internal medicine for women suffering from recurrent pregnancy loss (RPL). Specifically, our objectives were to (i) assess the impact of the 2023 ACR/EULAR APS classification criteria on the prevalence of APS in patients with RPL; and (ii) analyze the prevalence of RD within this patient population.

## 2. Materials and Methods

### 2.1. Participants

We conducted a retrospective longitudinal cohort study in the Department of Internal Medicine and Clinical Immunology of Rennes University Hospital, a French tertiary care center. According to the Royal College of Obstetricians and Gynaecologists (RCOG) guidelines, RPL was defined as three consecutive pregnancy losses before 14 WG [5]. We performed an exhaustive search in the hospital electronic database EHOP Clinical Data Warehouse, Rennes University Hospital, France [28] to retrieve all patients who were referred to the Internal Medicine and Clinical Immunology department for RPL from January 2010 until study onset in December 2021. The inclusion period began in January 2010, coinciding with the generalized implementation of APS testing in RPL patients in our center, using standardized methods. This decision was made to enhance the robustness of results and ensure better interindividual comparability.

Inclusion criteria included a complete negative RPL investigation according to current guidelines [5], with normal cytogenetic analysis of the conception product when available, normal parental peripheral blood karyotyping, normal pelvic ultrasound, and complete antiphospholipid testing. Patients with chromosomal abnormalities, with uterine malformations, or who were aged < 18 were not included.

Ethics approval was obtained from the Rennes Hospital Ethics Committee (authorization No. 20.164, approval date: 1 May 2021). This research was conducted in accordance with the institution’s protocol under the Methodology of Reference MR-004 (institutional registration number: 2205295 v 0). All participants provided informed consent.

### 2.2. Data Collection

Data were retrospectively extracted from patients’ electronic medical records. Baseline data were recorded at the time of medical consultations, while follow-up data were collected at the time the study was conducted. Patients were followed by their referring gynecologists across various hospitals within the district. Follow-up information, including details on treatments, fetal echography results, gestational age at birth, and pregnancy outcomes, was systematically collected from medical and birth records, which were routinely sent to the study center after consultations.

### 2.3. Study Variables

Data included age, ethnicity [29], tobacco use, BMI, gravidity, parity, and results of the RPL investigation. Patients’ obstetrical history included the following: number and term of miscarriages, fetal deaths, restriction growth, stillbirth or perinatal death, premature birth, pre-eclampsia, HELLP syndrome, ultrasonographic signs of placental vasculopathy, arterial or venous thrombosis during pregnancy, and results of placental analyses and fetal examination when available for each pregnancy. Placental analyses were all performed by the same specialized pathologist, who reported the following entities according to current guidelines: maternal vascular malperfusion (MVM), fetal vascular malperfusion (FVM), delayed villous maturation, and patterns of ascending intrauterine infection and villitis [30]. The composite variable “placental vascular pathology” included the following: history of pre-eclampsia, HELLP syndrome, in utero fetal growth restriction (IUFGR), and maternal vascular malperfusion (MVM) [30,31]. Clinical signs of RD were collected, including photosensitivity, joint pains, sicca syndrome, livedo, Raynaud’s phenomenon, and organ involvement including kidney insufficiency. The diagnosis of RD was determined by the clinician’s judgment during the consultation and documented in the patients’ records.

Antinuclear antibody (ANA) detection was performed via indirect immunofluorescence (IIF) on HEp-2 cells (Inova/Werfen) with initial screening dilutions of 1/80 and 1/160. If positivity exceeded 1/160, additional dilutions were performed up to 1/2560. Solid-phase assay for anti-ENA antibodies (SSA, TRIM21, SSB, RNP, Sm, Scl70, Jo-1, and Centromere) and double-stranded DNA (dsDNA) antibody test using ELIA on the Phadia250 platform (Phadia) were subsequently performed. In cases in which anti-ENA screening test was positive, specific ELIA unit tests were performed and confirmed by immunodot assays (Fujirebio). Lastly, the positivity of anti-dsDNA antibodies was confirmed, when applicable, via ELISA using the FarZyme test (Inova/Werfen).

aPLA testing was performed in the same laboratory from Rennes University Hospital. APS research included testing for the presence of LA, immunoglobulin IgG/IgM aCL, and IgG/IgM aB2GPI antibodies. LA was detected using diluted Russell’s viper venom and diluted activated partial thromboplastin time, according to the International Society for Thrombosis and Haemostasis (ISTH) recommendations [32]. aCL and aB2GPI antibodies were tested using colored microsphere-based flow cytometric assay (FIDIS APS, Theradiag, Croissy Beaubourg, France) from the time of the study’s implementation to December 2018. ELISA was used for patients tested after January 2019. Positive tests with colored microsphere-based flow cytometric assay were confirmed by ELISA according to guidelines [32]. Only the patients with confirmed positivity by ELISA testing were considered aPLA-positive in this study. For Sydney APS criteria, aPLA positivity was considered for titers above the 99th percentile of the values obtained for a normal population according to guidelines [32]. The thresholds of 40 and 80 units were used to classify patients according to the updated ACR/EULAR 2023 recommendations [7]. aPLA was considered positive only when positive tests were confirmed 12 weeks apart.

### 2.4. ACR/EULAR 2023 APS Classification Criteria

The 2023 antiphospholipid syndrome (APS) classification criteria use a refined scoring system, integrating a clinical domain and a laboratory domain, to enhance diagnostic precision. The clinical domain includes thrombotic events (arterial, venous, or small vessel), pregnancy morbidity, and specific neurological or cardiovascular manifestations. The updated criteria broaden the recognition of thrombosis at non-traditional sites, which were previously underrepresented. Stricter definitions have been introduced for pregnancy-related criteria, especially regarding the timing of pregnancy loss and complications such as pre-eclampsia. Additionally, the criteria now explicitly acknowledge microvascular involvement and cardiac valve disease, extending the clinical spectrum of APS. The laboratory domain focuses on aPLA serology, including LA, aCL, and aB2GPI. A significant update is the focus on medium (≥40 units) to high (≥80 units) titers for aCL and aB2GPI antibodies, enhancing diagnostic accuracy by excluding low titers that were previously acceptable under the Sydney criteria. Both domains are weighted according to the severity and relevance of the findings, and a combined score is generated. To classify as APS, a score of ≥3 points is required for both clinical and laboratory domains [7].

### 2.5. Study Design

Patients were separated in subgroups, according to aPLA status and after applying both classification criteria for APS. “aPLA negative” and “aPLA positive” groups were based on the positivity of aPLA at the 99th percentile, as per the Sydney criteria. Among aPLA-positive patients, two subgroups were defined based on whether they fulfilled only the Sydney criteria (sAPL) or met the updated criteria (uAPL). Finally, patients who did not fulfill the updated criteria for APS were identified as having experienced uRPL (unexplained recurrent pregnancy loss).

### 2.6. Statistical Analysis

Qualitative variables are presented with numbers and percentages. Quantitative variables are expressed with median (Q1–Q3). Qualitative data associations were analyzed by conducting the chi-squared test or Fisher’s exact test as appropriate. Variable distribution was assessed using the Shapiro–Wilk test. Data were analyzed by conducting the Student’s *t*-test or Mann–Whitney U test depending on Gaussian distribution. The McNemar test was used to analyze the effect of LMWH on live birth rate in patients’ subsequent pregnancies. Logistic regression was used to compare treatment effects between groups. Statistical analyses were performed using R software (v 3.6.0).

## 3. Results

**Figure 1:**
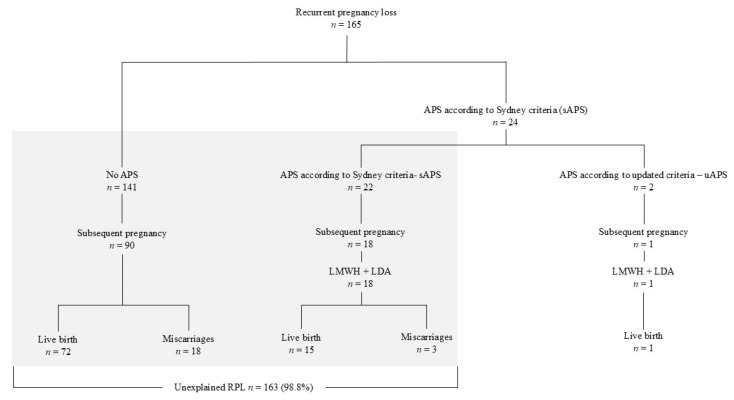
Study flow chart.

Over the study period, 289 patients underwent consultations for RPL. Among them, 165 met the inclusion criteria and were therefore included in the study (Figure 1). The patient characteristics are shown in Table 1.

Chronic diseases or particular conditions were found in 27 (16.4%) patients, including hypothyroidism (*n* = seven), rheumatoid arthritis (*n* = three), endometriosis (*n* = two), epilepsy (*n* = two), depression (*n* = four), diabetes (*n* = two), asthma (*n* = two), HIV (*n* = 1), sickle cell anemia (*n* = one), hereditary hemochromatosis (*n* = one), kidney transplant (*n* = 1), generalized anxiety disorder (*n* = one), and multiple sclerosis (*n* = one). For all treated patients, the LMWH dosage was 4000 IU/day, and the LDA dosage was 100 mg/day.

### 3.1. Immunological Features and Systemic Diseases

Antinuclear antibody (ANA) positivity > 1/160 was found in 46 patients (28%), among whom 24 (14%) had ANA > 1/320. One patient had anti-dsDNA with the anti-Ro/SSA52 antibody and the anti-Ro/SSA60 antibody. One patient had the anti-DFS70 antibody (anti-dense fine speckled 70 kDa), and one patient only had the ACPA (anti-citrullinated protein antibody).

RDs other than APS were newly diagnosed in three patients, including systemic lupus erythematosus (*n* = 1), rheumatoid arthritis (*n* = 1), and Behçet’s disease (*n* = 1). One patient had preexisting rheumatoid arthritis. This resulted in a prevalence of non-APS RDs of 2.4%. All of these patients exhibited clinical signs of rheumatic disease at the time of the consultation. None of them had impaired organ function, including renal failure.

### 3.2. Antiphospholipid Positivity

Twenty-four (14.5%) patients had a persistent positive aPLA serology according to the Sydney criteria and were classified within the “Sydney APS group” (sAPS). Among sAPS patients, two (8.3%) tested positive for LA, while 18 (75%) were positive for aCL and 18 (75%) for aB2GPI antibodies. Double positivity was observed in 11 patients (46%), and one patient exhibited triple positivity. Five patients had medium titers of aCL and/or aB2GPI IgM/IgG, and two had high titers (Supplementary Material—Table S1). Four patients had a prior history of arterial or venous thrombosis; however, each event was associated with a significant provoking factor. Furthermore, aPLAs tested negative at the time of thrombotic events, so these patients were not classified as having thrombotic APS (Supplementary Material—Table S2). aPLAs were negative in 141 (85.5%) patients, who were categorized as the “no-APS” group.

There was no significant difference regarding thromboembolic disease or obstetrical history including placenta vascular pathology between the “sAPS” and the “no-APS” groups. Joint pain was more frequently reported in the “sAPS” patients (17% vs. 4.3%, *p* = 0.040) (Table 1).

When applying the 2023 ACR/EULAR criteria to the 22 sAPS patients, 16 did not meet both the clinical and biological criteria. Five patients fulfilled the biological criteria but only had miscarriages, which did not reach the clinical threshold score. One patient met the clinical criteria but did not fulfill the biological criteria. Overall, 17 out of 22 sAPS patients (77%) did not meet the laboratory classification criteria under the updated guidelines. This resulted in only two patients from the “sAPS” group still being classified as having “APS”, who were included in an updated “uAPS” group. The mean clinical classification score in the “sAPL only” patients was 1.27, and the mean biological score was 1.32. Among the patients fulfilling the updated 2023 APS criteria, the mean clinical score was 3.5, and the mean biological score was 4. We did not find any difference between the sAPS patients and the two patients fulfilling the updated ACR/EULAR criteria (uAPS group).

### 3.3. Outcome of Subsequent Pregnancy

Overall, 109 (66.1%) patients had a new pregnancy after their consultations, including ninety (63.8%) patients in the “no-APS” group, nineteen (79.2%) in the “sAPS” group, and one in the “uAPS” group (Figure 1). All of the 19 “sAPS” patients received LDA + LMWH, resulting in 16 (80%) live births. The three patients with recurrent miscarriages showed a higher multigravidity (≥six previous pregnancies). The live birth rate was similar between the “no-APS” and “sAPS” patients (80% vs. 84%, *p* = 0.76) (Table 1). The effect of LMWH was assessed by comparing the intra-individual live birth rate, before and after the prescription of the LMWH. A significant live birth rate improvement was found in the “sAPL” and “no-APS” patients (*p* < 0.001), but the treatment effect did not differ between groups (*p* = 0.8). One of the two patients fulfilling the updated APS criteria had a subsequent pregnancy and received a combination of LMWH and LDA, resulting in a live birth.

Among the 163 uRPL patients, 108 had a new pregnancy, resulting in 87 (81%) live births and 21 (19%) miscarriages (Table 2). We did not find any association between study variables, including ANA or aPLA positivity, obstetric history (including the number of previous miscarriages), LMWH treatment, and pregnancy outcome.

In the uRPL group, 63 patients received LDA + LMWH, 31 received LDA only, and 14 had no treatment (Table 3). A higher frequency of aPLA positivity (*p* < 0.001) was found in the patients who received LMWH (Table 4). The live birth rate did not differ between patients receiving LMWH and those who did not receive any treatment (83% vs. 78%, respectively; *p* = 0.5). The gestational age at delivery in LMWH patients was one week less than those without LMWH (*p* < 0.02) and was related to labor induction in sAPS patients according to guidelines.

Among the 63 uRPL patients who received the LDA + LMWH combination for their next pregnancy, there was no difference in the prevalence of aPLA positivity (LA or aPL > 99th percentile) between patients with live births and those with recurrent miscarriages (28 vs. 27%, *p* > 0.9) or in terms of the prevalence of a history of placental vascular pathology (31 vs. 18%, *p* = 0.5). A higher mean gravidity was found in the patients with recurrent miscarriages as compared to those without (6 (5, 6.5) vs. 5 (4, 6); *p* = 0.05). (Table 4).

## 4. Discussion

This study, conducted at a tertiary referral center, provides new insights into the prevalence and clinical significance of rheumatic diseases (RDs), particularly antiphospholipid syndrome (APS), among patients with recurrent pregnancy loss (RPL). APS was diagnosed in 14% of the RPL patients using the Sydney criteria but in only 1.2% under the updated 2023 ACR/EULAR criteria. The overall prevalence of RD in this population was 17%, with only a small fraction (1.8%) of patients receiving new RD diagnoses unrelated to APS during their evaluation. Notably, no significant clinical or obstetrical differences were observed between patients with APS under the Sydney criteria and those without APS. These results challenge the association between oAPS, as defined by the Sydney criteria, and RPL and highlight the implications of the updated classification criteria for clinical practice and research.

Only three patients with RPL were newly diagnosed with RDs other than APS during their consultations. Although many studies have shown the higher risk of RPL in patients with RD, especially SLE [33], these results suggest that recurrent miscarriages are rarely the first manifestation of RD. Using the Sydney classification criteria, oAPS was found in 24 patients (14%). This is consistent with previous studies with an aPLA prevalence among RPL patients ranging from 5 to 20% [25,34]. Although the aPLA titers in our APS patients were relatively low, the distribution of aPLA profiles was consistent with findings from previous studies [35,36]. Due to our limited population size, we could not identify an association between biological profile, especially LA and aPLA triple positivity, and subsequent pregnancy prognosis [25,26].

Several findings from the present study challenge the association between RPL and oAPS as defined by the Sydney criteria. Firstly, we found no clinical differences between sAPS and no-APS patients, regarding either their obstetrical or clinical history. The prevalence of clinical signs frequently associated with APS, such as livedo or a history of thromboembolism, did not differ between the sAPS and no-APS patients. This is consistent with several data underscoring the phenotypic and prognostic differences between obstetrical and thrombotic APS [37].

The pathogenesis of aPLA during pregnancy involves endothelial dysfunction, resulting in placental vascular malperfusion [38,39]. We did not find a more frequent history of placental vascular lesions in the sAPS patients, as compared to the no-APS patients. While these findings further challenge the association between sAPS and RPL, they also suggest that exploring other immunological pathways linked to APLA toxicity during pregnancy is needed. Exploring the role of complement pathway activation on trophoblast cells’ cytotoxicity and endothelial dysfunction may help us to better understand the potential role of aPLA in early miscarriages [40,41,42].

Thirdly, the effectiveness of combining LMWH and LDA in RPL patients with oAPS remains uncertain, as previous randomized controlled trials have provided limited evidence for the efficacy of LDA ± LMWH in this subgroup [21,43,44,45]. In our study, aPLA positivity was not associated with better subsequent pregnancy outcomes in the patients receiving a combination of LMWH and LDA. Moreover, the intra-individual effect of LMWH on subsequent pregnancy outcome did not differ between the aPLA-negative patients and sAPS patients as per the Sydney criteria. While our study highlights the positive outcomes of subsequent pregnancies in sAPS patients, sAPS as per the Sydney criteria was not a predictor of an improved response to LDA/LMWH.

The limited evidence supporting a causal relationship between aPLA positivity and RPL led to reducing the weight of these manifestations within the updated clinical criteria. When applying these criteria in our population, the prevalence of oAPS largely decreased from 24 (14%) to 2 (1.21%) patients. Such a small sample limited direct statistical comparisons between uAPS, sAPS, and no-APS patients. This finding is consistent with a recent study by Foddai et al., which showed that in an international cohort, 48.7% of patients previously classified as APS according to the Sydney criteria failed to meet the ACR/EULAR 2023 criteria [46]. This discrepancy was especially notable in the oAPS subgroup, in which the prevalence dropped from 26.9% to 3.2% under the new criteria. Notably, in our study, none of the patients classified as oAPS based on RPL under the Sydney criteria met the updated criteria, underscoring the limitations of the weight given to RPL in the clinical criteria domain of the updated criteria. The lack of standardization in APS testing methods and the variability in positivity thresholds were also emphasized during the development of the updated APS criteria. Consequently, the positivity thresholds were raised to 40 IU (intermediate level) and 80 IU (high level) in the new criteria. This resulted in 17/22 (77%) of the APS patients defined by the Sydney criteria in our study no longer meeting the biological classification criteria under the updated guidelines.

These updated thresholds and revised weightings of diagnostic criteria were developed to address the challenges in APS diagnosis, particularly the lack of standardization in testing methods and variability in positivity thresholds. The intention behind the changes is to improve the consistency and quality of research, by reducing false positives and ensuring a more homogenous patient population. Accordingly, a recent study demonstrated that raising the aPLA threshold significantly reduced the number of patients who were classified as having APS, particularly those with isolated aPLA positivity [46]. However, while these criteria offer better specificity, their transferability into clinical practice remains to be explored. A key limitation of the updated criteria is their reduced sensitivity, especially in patients presenting with early obstetric manifestations such as recurrent pregnancy loss (RPL). This raises concerns regarding the underdiagnosis of obstetric APS, particularly in the absence of thrombotic events, as the criteria now require more robust clinical and laboratory evidence for diagnosis. This shift reflects the ongoing debate about the role of aPLAs in pregnancy loss, with some studies suggesting that even low-titer aPLAs could contribute to RPL [47]. Consequently, while the updated APS criteria provide valuable improvements in research standardization and reduce diagnostic ambiguity, they also highlight the need for further exploration in clinical settings, particularly regarding early obstetric presentations like RPL. The 2023 ACR/EULAR guidelines explicitly acknowledge these uncertainties and call for further research to explore the nuanced relationship between aPLA antibodies and obstetric outcomes, which will be crucial for refining these criteria and their application in diagnosing and managing APS in clinical practice.

To that end, future studies are needed to compare the incidence of RPL between aPLA patients and healthy subjects to definitively determine the potential association between APS and RPL. Given the rare prevalence of APS according to the updated criteria, such studies would benefit from large, multicenter designs, which would provide sufficient statistical power and allow for subgroup analyses based on aPLA profiles. Whether APS is a rare disease was debated until recently [48]. Regarding obstetric APS, these new criteria resolve this controversy, reclassifying obstetric APS as a rare disease. Using these updated criteria with a higher specificity in future works will likely enable us to better assess the efficacy of the combination of LMWH and LDA. Such efforts will help explore the association between oAPS and RPL and help determine the best treatment regimen for these patients.

Conversely, applying the 2023 ACR/EULAR criteria resulted in classifying almost all patients as uRPL. In our study, we did not identify any differences between patients meeting only the Sydney criteria (sAPS) and non-APS patients. This supports the comparability between these populations and reinforces the new classification criteria, which do not recognize recurrent miscarriages as a manifestation related to APS. Subsequent pregnancies in these uRPL patients resulted in live births 80% of the time, which was consistent with previous studies [8,49,50]. We did not find any association between any study variables and subsequent pregnancy outcomes. These results could be explained by the high success rate in subsequent pregnancies, rendering our patient sample size too limited to achieve sufficient statistical power. Notably, aPLA positivity in patients with uRPL was not predictive of subsequent pregnancy outcomes. Although several data suggest a prognostic value of ANA positivity [51], we found no association between ANA positivity and the outcome of subsequent pregnancies. Currently, isolated ANA positivity is not known to influence the therapeutic options for women with RPL. Considering the low prevalence of newly diagnosed auto-immune diseases in our cohort, these findings question the relevance of the updated ESHRE 2022 guidelines, which recommend systematic ANA testing for all patients with RPL, even in the absence of any clinical signs of RD [52]. Hence, further research is needed to identify relevant prognostic biomarkers and to guide the effective management of future pregnancies in patients with unexplained recurrent pregnancy loss.

### Limitations

Our study was limited by its sample size, with only twenty-four oAPS patients included, and only two patients with oAPS as defined per the revised criteria, thus hindering us from performing multivariate analyses. Due to the limited number of patients with oAPS, the low frequency of LA positivity (*n* = 2), and the high live birth rates observed in subsequent pregnancies (84%), we did not identify a prognostic impact of the aPLA serological profile on subsequent pregnancy outcomes, although this was not the primary objective of the study.

This study was conducted at a single tertiary care center, with potential selection bias. However, this approach aimed at improving the homogeneity of the study population, ensuring that all patients underwent an exhaustive RPL work-up according to guidelines. Genetic analyses of conception products from previous or subsequent miscarriages were unavailable for most of the patients, though this reflects real-world practice.

The aPLA titers observed in our study were relatively low, although they are consistent with findings from previous studies [35,36]. This can be explained by the retrospective design of our study, which involved changes in the detection methods used, making comparisons with the new thresholds, particularly those in the updated classification, difficult. Under the Sydney classification, patients were considered positive if their aPLA titers were above the 99th percentile, which was the case in our cohort, further reflecting the stricter and more specific nature of the new biological criteria. As such, these results are representative of a real-world cohort of patients with recurrent miscarriages.

## 5. Conclusions

In a cohort of 165 women with recurrent pregnancy loss (RPL), antiphospholipid syndrome (APS) was diagnosed in 14% of the patients using the Sydney criteria, but this sharply decreased to just 1.2% with the updated ACR/EULAR 2023 criteria. The overall prevalence of autoimmune diseases was 17%, with only three cases of non-APS rheumatic diseases diagnosed, challenging the current practice of routine ANA testing in RPL patients. The absence of significant differences in pregnancy history, clinical features, or outcomes between APS (as per the Sydney criteria) and non-APS patients highlights the lack of specificity of the Sydney criteria—a limitation addressed by the ACR/EULAR 2023 criteria. However, the new criteria’s failure to capture a larger subset of RPL patients suggests that they may miss clinically relevant cases, limiting their clinical utility. These findings suggest that classification criteria may have limitations when used as diagnostic tools in routine practice. Therefore, diagnosis and treatment decisions should be based on a comprehensive assessment of each patient’s presentation, informed by the physician’s expertise and available scientific evidence. Furthermore, our results underscore the need for additional research to refine the criteria and optimize treatment strategies—such as the LMWH-LDA combination—based on more precise patient selection.

## Figures and Tables

**Table 1:** Comparison between the “no-APS” group and APS group according to Sydney criteria (sAPS).

Patient Characteristics	Overall *n* = 165	No-APS *n* = 141	sAPS *n*= 24	*p*-Value ^1^
Age, median (Q1–Q3), years	33.3 (29.9, 36.7)	33.1 (29.8, 36.7)	33.9 (30.6, 36.2)	0.74
Smoking history, *n* (%)	47 (28)	44 (31)	3 (12)	0.06
Ethnic group, *n* (%)				0.61
Caucasian	151 (92)	128 (91)	23 (96)	
Maghrebin	8 (4.8)	8 (5.7)	0 (0)	
Afro-Caribbean	5 (3.0)	4 (2.8)	1 (4.2)	
Asian	1 (0.6)	1 (0.7)	0 (0)	
Body mass index, median (Q1–Q3)	23.1 (21.0, 26.4)	24.1 (4.5)	24.3 (3.3)	0.37
Photosensivity, *n* (%)	1 (0.6)	1 (0.7)	0 (0)	>0.9
Joint pain, *n* (%)	10 (6.1)	6 (4.3)	4 (17)	0.040
Acrocyanosis, *n* (%)	10 (6.1)	10 (7.1)	0 (0)	0.36
Sicca syndrome, *n* (%)	2 (1.2)	2 (1.4)	0 (0)	>0.9
Livedo, *n* (%)	10 (6.1)	8 (5.7)	2 (8.3)	0.64
Raynaud’s phenomenon, *n* (%)	11 (6.7)	11 (7.8)	0 (0)	0.37
Thrombosis (arterial or venous), *n* (%)	6 (3.6)	4 (2.8)	2 (8.3)	0.21
Gestity, *n* (%)	5.00 (4.00, 6.00)	5.00 (4.00, 6.00)	4.00 (3.75, 6.00)	0.16
≥6	60 (36)	52 (37)	8 (33)	
<6	105 (64)	89 (63)	16 (67)	
Pregnancy, *n* (%)				
Live birth	73 (44)	65 (46)	8 (33)	0.24
Fetal death > 24 WG	14 (8.5)	12 (8.5)	2 (8.3)	>0.9
Intrauterine growth restriction	16 (9.7)	15 (11)	1 (4.2)	0.47
Placental vascular pathology	29 (18)	25 (18)	4 (17)	>0.9
HELLP or PE	6 (3.6)	5 (3.5)	1(4.2)	>0.9
Recurrent pregnancy loss only	75 (45)	63 (45)	12 (50)	0.63
ANA, *n* (%)				
≥1/80	75 (45)	60 (50)	15 (62)	0.11
≥1/160	46 (28)	40 (33)	6 (25)	0.92
≥1/320	24 (14)	20 (17)	5 (21)	0.37
Subsequent pregnancy	*n* = 109	n = 90	n = 19	
Live birth, *n* (%)	88 (79)	72 (80)	16 (84)	0.76
Miscarriage, *n* (%)	21 (13)	18 (20)	3 (16)	
Gestation time at delivery, median (Q1–Q3), weeks	38 (36,39)	39.00 (37.00, 40.00)	38.00 (37.00, 39.00)	0.30
Treatment, *n* (%)				
Treatment LDA + LMWH	64 (59)	45 (50)	19 (100)	<0.001
Treatment LDA	31(28)	31 (34)	0	0.19
No treatment	14 (13)	14 (16)	0	0.002

^1^ Wilcoxon rank sum test; Fisher’s exact test; Pearson’s chi-squared test. ANAs: Antinuclear antibodies; HELLP: Hemolysis, Elevated Liver enzymes and Low Platelets; LDA: Low-dose aspirin; LMWH: Low-molecular-weight heparin; PE: Pre-eclampsia; WG: Weeks of gestation.

**Table 2:** Comparative analysis of uRPL patients according to subsequent pregnancy outcome.

uRPL and Next Pregnancy N = 108	Live Birth *n* = 87	Miscarriage *n* = 21	*p*-Value ^1^
Age, median (Q1–Q3), years	31.7 (29.4, 35.2)	34.0 (32.0, 36.5)	0.15
Smoking history, *n* (%)	30 (34)	4 (19)	0.17
Ethnic group, *n* (%)			0.33
Caucasian	83 (95)	19 (90)	
Maghrebin	2 (2.3)	1 (4.8)	
Afro-Caribbean	2 (2.3)	1 (4.8)	
Asian	0 (0)	0 (0)	
Body mass index, median (Q1–Q3)	23.7 (3.9)	26.3 (6.2)	0.09
Photosensivity, *n* (%)	1 (1.1)	0 (0)	>0.9
Joint pain, *n* (%)	5 (5.7)	1 (4.8)	>0.9
Acrocyanosis, *n* (%)	6 (6.9)	0 (0)	0.59
Sicca syndrome, *n* (%)	0 (0)	0 (0)	
Livedo, *n* (%)	6 (6.9)	1 (4.8)	>0.9
Raynaud’s phenomenon, *n* (%)	5 (5.7)	0 (0)	0.58
Thrombosis (arterial or venous), *n* (%)	3 (3)	2 (9)	0.25
Gestity, *n* (%)	5.00 (4.00, 6.00)	5.00 (5.00, 6.00)	0.15
≥6	26 (30)	10 (48)	
<6	61 (70)	11 (52)	
Pregnancy, *n* (%)			
Live birth	35 (40)	10 (48)	0.54
Fetal death > 24 WG	7 (8.0)	2 (9.5)	>0.9
Intrauterine growth restriction	11 (13)	1 (4.8)	0.45
Placental vascular pathology	22 (25)	2 (9.5)	0.15
HELLP or PE	5 (5.7)	0 (0)	0.58
Recurrent pregnancy loss only	45 (52)	8 (28)	0.26
ANA, *n* (%)			
≥1/80	37 (48)	9 (43)	>0.9
≥1/160	23 (30)	7 (33)	0.71
≥1/320	17 (22)	3 (14)	0.76
aPLA positivity according to Sydney criteria, *n* (%)	15 (17)	3 (14)	>0.9
aPLA positivity according to updated criteria, *n* (%)	4 (5)	0 (0)	>0.9
No treatment	11 (13)	3 (14)	>0.9
LDA alone	24 (27)	7 (33)	0.79
LDA + LMWH	52 (60)	11 (52)	0.71

^1^ Wilcoxon rank sum test; Fisher’s exact test; Pearson’s chi-squared test. ANAs: Antinuclear antibodies; aPLAs: Antiphospholipid antibodies; HELLP: Hemolysis, Elevated Liver enzymes and Low Platelets; LDA: Low-dose aspirin; LMWH: low-molecular-weight heparin; PE: Pre-eclampsia; WG: Weeks of gestation.

**Table 3:** Comparative analysis of uRPL patients based on the treatment received during subsequent pregnancies.

uRPL and Subsequent Pregnancies N = 108	LMWH + LDA *n* = 63	Other Treatments *n* = 45	*p*-Value ^1^
Age, median (Q1–Q3), years	33 (29.7, 36.1)	31.3 (29.4, 33.8)	0.11
Smoking history, *n* (%)	20 (31)	14 (32)	>0.9
Ethnic group, *n* (%)			0.82
Caucasian	60 (95)	42 (93)	
Maghrebin	1 (1.6)	2 (4.4)	
Afro-Caribbean	2 (3.2)	1 (2.2)	
Body mass index, median (Q1–Q3)	23.1 (21.1, 25.7)	24.4 (20.8,27.0)	0.55
Photosensivity, *n* (%)	0	1 (2.2)	0.42
Joint pain, *n* (%)	4 (6.3)	2 (4.4)	>0.9
Acrocyanosis, *n* (%)	3 (4.8)	3 (6.7)	0.69
Sicca syndrome, *n* (%)	0	0	
Livedo, *n* (%)	5 (7.9)	2 (4.4)	0.70
Raynaud’s phenomenon, *n* (%)	4 (6.3)	1 (2.2)	0.40
Thrombosis (arterial or venous), *n* (%)	4 (6.3)	1 (2.2)	0.40
Gestity, *n* (%)	5 (4.00, 6.00)	5 (4.00,5.00)	0.11
≥6	25 (40)	11 (24)	
<6	38 (60)	34 (76)	
Pregnancy, *n* (%)			
Live birth	29 (46)	16 (36)	0.28
Fetal death > 24 WG	6 (9)	3 (6.7)	0.73
Intrauterine growth restriction	9 (14)	3 (6.7)	0.21
Placental vascular pathology	18 (29)	6 (13)	0.06
HELLP or PE	5 (7.9)	0	0.07
Recurrent pregnancy loss only	29 (46)	24 (53)	0.45
ANA, *n* (%)			
≥1/80	27 (44)	18 (40)	0.71
≥1/160	16 (26)	13 (29)	0.72
≥1/320	10 (16)	9 (20)	0.60
aPLA positivity according to Sydney criteria, *n* (%)	18	0	<0.001
aPLA positivity according to updated criteria, *n* (%)	0	0	-
Subsequent pregnancy			
Live birth	52 (83)	35 (78)	0.54
Miscarriage	11 (17)	10 (22)	0.54
Gestation time at delivery, median (Q1–Q3), weeks	38 (37.00, 39.00)	39 (38.00, 40.00)	0.017

^1^ Median (Q1–Q3; n (%). Wilcoxon rank sum test; Fisher’s exact test; Pearson’s chi-squared test. ANAs: Antinuclear antibodies; aPLAs: Antiphospholipid antibodies; HELLP: Hemolysis, Elevated Liver enzymes and Low Platelets; LDA: Low-dose aspirin; LMWH: Low-molecular-weight heparin; PE: Pre-eclampsia; WG: Weeks of gestation.

**Table 4:** Comparison of uRPL patients receiving LMWH and LDA according to subsequent pregnancy outcome.

uRPL Patients Receiving LMWH + LDA N = 63	Live Birth *n* = 52	Miscarriage *n* = 11	*p*-Value ^1^
Age, median (Q1–Q3), years	32.5 (4.6)	34.1 (3.6)	0.22
Smoking history, *n* (%)	19 (37)	1 (9.1)	0.15
Ethnic group, *n* (%)			
Caucasian	49 (94)	11 (100)	>0.99
Maghrebin	1 (1.9)	0 (0)	
Afro-Caribbean	2 (3.8)	0 (0)	
Asian	0 (0)	0 (0)	
Body mass index, median (Q1–Q3)	23.9 (4.2)	23.9 (3.2)	0.81
Photosensivity, *n* (%)	0 (0)	0 (0)	
Joint pain, *n* (%)	3 (5.8)	1 (9.1)	0.55
Acrocyanosis, *n* (%)	3 (5.8)	0 (0)	>0.9
Sicca syndrome, *n* (%)	0 (0)	0 (0)	
Livedo, *n* (%)	4 (7.7)	1 (9.1)	>0.9
Raynaud’s phenomenon, *n* (%)	4 (7.7)	0 (0)	>0.9
Thrombosis (arterial or venous), *n* (%)	3 (5.8)	1 (9.1)	0.55
Gestity, *n* (%)	5.00(4.00, 6.00)	6.00 (5.00, 6.50)	0.050
≥6	18 (35)	7 (64)	
<6	34 (65)	4 (36)	
Pregnancy, *n* (%)			
Live birth	23 (44)	6 (55)	0.53
Fetal death > 24 WG	5 (9.6)	1 (9.1)	>0.9
Intrauterine growth restriction	8 (15)	1 (9.1)	>0.9
Placental vascular pathology	16 (31)	2 (18)	0.49
HELLP or PE	5 (9.6)	0 (0)	0.58
Recurrent pregnancy loss only	25 (48)	4 (36)	0.52
ANA, *n* (%)			
≥1/80	23 (49)	5 (55)	>0.9
≥1/160	14 (30)	3 (33)	>0.9
≥1/320	10 (21)	1 (11)	0.67
aPLA positivity according to Sydney criteria, *n* (%)	15 (28)	3 (27)	>0.9
aPLA positivity according to updated criteria, *n* (%)	4 (8)	0 (0)	>0.9

^1^ Wilcoxon rank sum test; Fisher’s exact test; Pearson’s chi-squared test. ANAs: Antinuclear antibodies; aPLAs: Antiphospholipid antibodies; HELLP: Hemolysis, Elevated Liver enzymes and Low Platelets; LDA: Low-dose aspirin; LMWH: Low-molecular-weight heparin; PE: Pre-eclampsia; WG: Weeks of gestation.

## Data Availability

Data supporting this study can be made available from the authors upon reasonable request.

## Supplementary Materials

The following supporting information can be downloaded at: https://www.mdpi.com/article/10.3390/jcm13247698/s1, Table S1: Description of aPLA profile in the study population; Table S2: Description of thrombotic events in the study population.

## Abbreviations

aB2GPI	anti-B2 glycoprotein 1
aCL	anti-cardiolipin
ACOG	American College of Obstetricians and Gynecologists
ACPA	Anti-citrullinated protein antibodies
ANA	Antinuclear antibody
Anti-DFS 70	Anti-dense fine speckled 70 kDa antibodies
Anti ds-DNA	Anti-double-stranded DNA antibodies
Anti-ENA	Anti-early nuclear antigens antibodies
aPLA	Anti-phospholipid antibodies
APS	Anti-phospholipid syndrome
CNGOF	Collège National des Gynécologues Obstétriciens Français
HELLP	Hemolysis, Elevated Liver enzymes and Low Platelets
FVM	Fetal vascular malperfusion
IgM	Immunoglobulin M
IgG	Immunoglobulin G
ISTH	International Society for Thrombosis and Haemostasis
IUFD	Unexplained intrauterine fetal death
IUGFR	Intrauterine growth fetal retardation
LA	Lupus anticoagulant
LDA	Low-dose aspirin
LMWH	Low-molecular-weight heparin
MVM	Maternal vascular malperfusion
PE	Pre-eclampsia
oAPS	Obstetric anti-phospholipid syndrome
RCOG	Royal College of Obstetricians and Gynaecologists
RD	Rheumatic disease
RPL	Recurrent pregnancy loss
sAPS	Antiphospholipid syndrome according Sydney criteria
SLE	Systemic lupus erythematosus
uAPS	Antiphospholipid syndrome according to EULAR/ACR 2023 criteria
uRPL	Unexplained recurrent pregnancy loss
VTE	Venous thromboembolism
WG	Weeks of gestation
