# Supplementary material for: Prevalence of Antiphospholipid Antibody Syndrome Among Patients with Recurrent Pregnancy Loss: Impact of the Revised 2023 ACR/EULAR Antiphospholipid Syndrome Criteria
Source: J Clin Med. 2024 Dec 17;13(24):7698. doi: 10.3390/jcm13247698 (PMC11677686; doi:10.3390/jcm13247698)
Supplement: Supplementary file 1 [file jcm-13-07698-s001.zip › jcm-3332947-supplementary.pdf]

Table S1. Description of aPLA profile in the study population.

Characteristic (*n* , %)	aPLA serology							
	LA	AcL IgG	AcL IgM	aB2GPI IgG	aB2GPI IgM	Single +	Double +	Triple +
Whole cohort *n*=165	2 (1,2)	11 (6)	13 (7)	12 (7)	9 (5)	12 (7)	11 (6,6)	1 (0,6)
oAPS (Sydney criteria) group *n*=24 (14%)	2 (8,3)	11 (45,8)	13 (54)	12 (50)	9 (37,5)	12 (50)	11 (45,8)	1 (4,1)
uAPS (2023 ACR/EULAR criteria) group *n*=2 (1.2%)	2 (100)	1 (50)	1 (50)	2 (100)	1 (50)	0	1 (50)	1 (50)
Median titer (U/mL)	NA	66	58	179	59			
Min-Max (U/mL)	NA	41-83	41- 146	69-406	49-134			

aB2GPI: anti-B2 glycoprotein 1 antibodies; aCL: anti-cardiolipin antibodies; LA: lupus anticoagulant; oAPS: Obstetric anti-phospholipid syndrome; uAPS: Antiphospholipid syndrome according to EULAR/ACR 2023 criteria

Table S2. Description of the thrombotic events in the study population

Patient	Arterial or venous thrombosis	Provoked thromboembolism	aPLA serology	Time between thrombosis and aPLA test
1	Deep venous thrombosis	No	Negative	1 month after event
2	Pulmonary embolism	No	Negative	At thrombosis event
3	Immediate postpartum pulmonary embolism	Yes	Negative	At thrombosis event
4	Deep Venous thrombosis on the 15th day of the postpartum period	Yes	Negative	At thrombosis event
